# Production and Mechanical Characterisation of TEMPO-Oxidised Cellulose Nanofibrils/β-Cyclodextrin Films and Cryogels

**DOI:** 10.3390/molecules25102381

**Published:** 2020-05-20

**Authors:** Bastien Michel, Julien Bras, Alain Dufresne, Ellinor B. Heggset, Kristin Syverud

**Affiliations:** 1 Univeristy Grenoble Alpes, CNRS, Grenoble INP*, LGP2, 38000 Grenoble, France; bastien.michel@lgp2.grenoble-inp.fr (B.M.); julien.bras@grenoble-inp.fr (J.B.); alain.dufresne@grenoble-inp.fr (A.D.); 2 RISE PFI, NO-7491 Trondheim, Norway; ellinor.heggset@rise-pfi.no; 3 Departments of Chemical Engineering, NTNU, 7491 Trondheim, Norway

**Keywords:** nanocellulose, β-cyclodextrin, cryogels, films

## Abstract

Wood-based TEMPO-oxidised cellulose nanofibrils (toCNF) are promising materials for biomedical applications. Cyclodextrins have ability to form inclusion complexes with hydrophobic molecules and are considered as a method to bring new functionalities to these materials. Water sorption and mechanical properties are also key properties for biomedical applications such as drug delivery and tissue engineering. In this work, we report the modification with β-cyclodextrin (βCD) of toCNF samples with different carboxyl contents viz. 756 ± 4 µmol/g and 1048 ± 32 µmol/g. The modification was carried out at neutral and acidic pH (2.5) to study the effect of dissociation of the carboxylic acid group. Films processed by casting/evaporation at 40 °C and cryogels processed by freeze-drying were prepared from βCD modified toCNF suspensions and compared with reference samples of unmodified toCNF. The impact of modification on water sorption and mechanical properties was assessed. It was shown that the water sorption behaviour for films is driven by adsorption, with a clear impact of the chemical makeup of the fibres (charge content, pH, and adsorption of cyclodextrin). Modified toCNF cryogels (acidic pH and addition of cyclodextrins) displayed lower mechanical properties linked to the modification of the cell wall porosity structure. Esterification between βCD and toCNF under acidic conditions was performed by freeze-drying, and such cryogels exhibited a lower decrease in mechanical properties in the swollen state. These results are promising for the development of scaffold and films with controlled mechanical properties and added value due to the ability of cyclodextrin to form an inclusion complex with active principle ingredient (API) or growth factor (GF) for biomedical applications.

## 1. Introduction

Cellulose nanofibrils (CNFs) are high-aspect ratio nanoparticles formed by bundles of cellulose chains that are a succession of glucose subunits linked by β-1-4 glycosidic bonds. CNFs are produced from a cellulosic raw material, usually wood, the most abundant and renewable polymer available on earth. CNFs are produced by a combination of chemical/enzymatic pretreatments and mechanical treatment, usually using a homogeniser [1], a microfluidiser [2], or a grinder [3]. The variety of existing pretreatments [4] allows for a variety of surface chemistries, making CNF materials suitable for many applications [5]. CNFs pretreated in the presence of (2,2,6,6-tetramethyl-piperidin-1-yl)oxyl, also known as TEMPO, proposed by Saito et al., 2006, which consists of the regioselective oxidation of C6 primary hydroxyls of cellulose to C6 carboxylate groups, have been considered in a wide variety of applications due to its carboxyl content and reduced size [6,7]. CNFs are generally used in two different forms: either as films/nanopapers or as gels and can be used as rheology modifiers or emulsion stabilisers and additives in many applications. Films are obtained by solvent casting [8,9,10], and nanopapers are obtained by filtration [11,12]. Three types of gels can be identified: hydrogels, cryogels obtained by freeze-drying, and aerogels obtained by supercritical drying [13,14]. For many applications, water sorption properties are important. The TEMPO-oxidised cellulose nanofibrils (toCNF), like cellulose, are hygroscopic materials, which means that they can attract and retain water molecules from their environment by absorption or adsorption [10]. The impact of process parameters on cryogels mechanical properties have been previously studied [14,15,16,17,18,19], highlighting the importance of density and preparation method on the mechanical properties.

As a natural, biodegradable, and abundant polymer with reactive surface chemistry and good biocompatibility, nanocellulose is a promising material within the medical field. In recent years, applications in wound healing [20,21], drug delivery [9], and tissue engineering [22] have been investigated. In tissue engineering, the scaffold should stimulate cells to differentiate, proliferate, and form tissue. The interplay between the matrix and cells should be driven by the action of signals, which can be a mechanical stimulation, chemical compounds, or growth factors (usually proteins) [23]. To be suited for tissue engineering applications, scaffolds needs to exhibit stiffness similar to the natural extra-cellular matrix (ECM) of the tissue to be repaired and measured by the elastic modulus E. Typical values of stiffness of ECM are 0.1–1 KPa for brain tissue, 8–17 KPa for muscle tissue, and 25–40 KPa for the cross-linked collagen matrix [24,25]. Another crucial aspect for tissue engineering application is the scaffold architecture. A high porosity is needed to promote the cellular penetration and an adequate diffusion of nutrients to the cells [26].

The utilisation of wood-based CNFs for tissue-engineering applications is encouraged by recent studies that confirmed the safety of CNFs [27,28,29,30], the construction of cell-friendly porous structures [14], and the control of mechanical properties [31,32]. In addition, CNFs, in the form of a highly entangled network, have shown the ability to retain the active principle ingredient up to several months [9]. However, major challenges for biomedical applications are yet to be overcome, such as increasing the bioavailability of drugs, as most new drugs are described as poorly soluble [33], and controlling the delivery kinetics of active principle ingredient (API). To address these issues, this research study proposes the use of cyclodextrins (CD).

Cyclodextrins are cyclic oligosaccharides consisting of glucose subunits linked by α-1-4 glycosidic bonds. Due to their conformation, with a hydrophobic interior and a hydrophilic exterior, these macromolecules exhibit cage-like properties and can form an inclusion complex with hydrophobic compounds [34,35]. These properties have led to their use in various fields, such as cosmetics, food, environment, and medicine [36,37,38]. Regarded as safe, they are widely used as an excipient in the pharmaceutical field [39,40]. For such applications, β-cyclodextrin (βCD), a cyclodextrin with seven glucose subunits, and its derivatives are the most commonly used [33,41]. βCD are also of a great interest for tissue engineering applications, with their properties to encapsulate lipophilic compounds proven to improve the performance of scaffolds [42,43,44]. This property could also lead to the immobilisation of the growth factor [45] or drug delivery [39] during the cell growth to optimise the effect of the scaffold. Previous studies reported the association of cyclodextrin with various cellulose derivatives [46,47]. The association between CDs and CNFs or cellulose nanocrystals (CNCs) has been attempted in a very few and recent studies, summarised in Table 1. To the best of our knowledge, no study presents the impact of βCD on both the sorption and the mechanical properties of toCNF substrates (films or cryogels).

The aim of the present study is to modify toCNF with βCD (preferably with covalent linkage) and to see what kind of effect this surface functionalisation has on the sorption and mechanical properties. Thus, a comparison with the same structures using unmodified toCNF is necessary. For that purpose, two suspensions of toCNF with different charge contents were prepared. Fibre modification with cyclodextrins was carried out at neutral and acidic (pH 2.5) to study the effect of the dissociation of the carboxylic acid group. Films, processed by casting/evaporation at 40 °C and cryogels, processed by freeze-drying were prepared from βCD-modified toCNF and compared with reference samples of unmodified toCNF. Water sorption was evaluated gravimetrically for both films and cryogels. The impact of density on the mechanical properties of the cryogels was assessed for cryogels obtained from unmodified toCNF and prepared by freeze-drying from suspensions at different dry matter contents for both charge contents. Compression tests in the dry and swollen state were performed on cryogels from all suspensions, and microscopic observation (SEM) was carried out to link the mechanical behaviour to the macroscopic structure of the materials.

## 2. Results and Discussion

TEMPO-oxidised cellulose nanofibril suspensions were successfully produced. The amounts of carboxylic groups were determined to be 756 ± 4 µmol/g and 1048 ± 32 µmol/g. Films and cryogels were processed from the two different toCNF suspensions in four different conditions presented in Table 2. 

**Figure 1:**
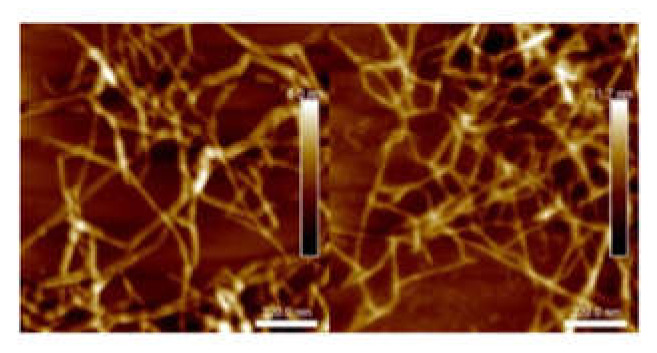
Atomic force microscopy (AFM) images of L-toCNF (left) and H-toCNF (right). toCNFs: TEMPO-oxidised cellulose nanofibrils. L and H are low and high contents, respectively.

The suspension with 756 µmol/g carboxyl content will be referred as L-toCNF in the text; hence, samples from L-toCNF will be labelled L1, L2, L3, and L4. Similarly, the suspension with 1048-µmol/g carboxyl content will be referred as H-toCNF; i.e., samples from H-toCNF will be labelled H1, H2, H3, and H4. Figure 1 shows atomic force microscopy (AFM) pictures of the nanofibers obtained for both charge contents. Similar and slightly thinner fibrils were obtained for the most oxidised cellulose, as expected.

### 2.1. Water Sorption Analysis

#### 2.1.1. Films

**Figure 2:**
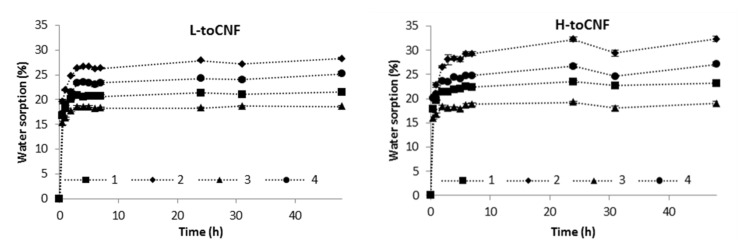
Water sorption as a function of time of conditioning at 25 °C 90% relative humidity (RH) for L-toCNF (left) and H-toCNF (right).

Water sorption of films was assessed gravimetrically in a Percival climatic chamber at 25 °C and 90% relative humidity (RH) for 48 h. Figure 2 displays the time dependence of water sorption for films with various carboxyl contents and casting conditions (casting pH and amount of cyclodextrin). Sorption equilibriums and % of sorption equilibriums after 30 min are reported in Table 3.

**Figure 3:**
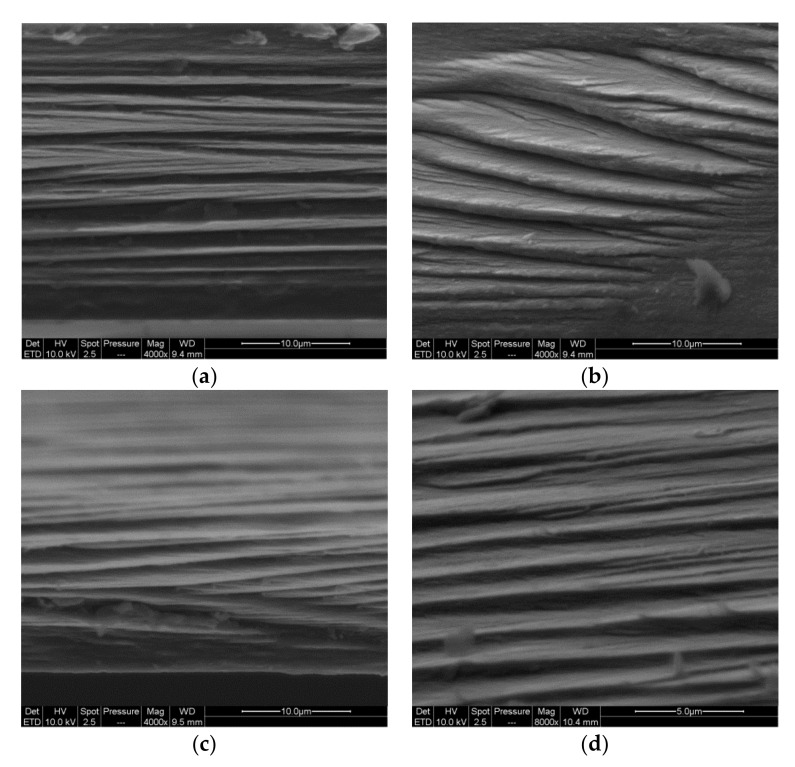
SEM images of the cross-section of films casted from suspensions H1 (**a**), H2 (**b**), H3 (**c**), and H4 (**d**).

For each sample of both L-toCNF and H-toCNF, the sorption equilibrium was reached after approximately 4 h, which indicates that this property is not dependent of any of the variable parameters in this study. Table 3 reports the sorption equilibrium obtained after 48 h. It is slightly higher for H-toCNF samples compared to L-toCNF: + 1.9% for raw suspension samples (condition 1), + 4% for pH 2.5 samples (condition 2), + 0.4% for 10 wt% CD samples (condition 3), and + 1.9% for 10 wt% CD/pH 2.5 samples (condition 4). For both charge contents, the same dynamic between the different casting conditions was observed: sample 3 < 1 < 4 < 2. The carboxylic content at acidic pH is in its carboxylic acid form, hence increasing the water sorption by forming more H-bound with water molecules than in neutral conditions. Cyclodextrins, by adsorbing onto the toCNF fibres, decreases the water sorption ability. For hygroscopic cellulosic materials, the two mechanisms of sorption, namely adsorption and absorption, need to be considered. In addition, depending on the surface chemistry of the fibres, sorption can be either slow or fast [59]. In the case of toCNF, due to their hydroxyl and carboxylic surface groups, sorption occurs quite fast. We can distinguish also two mechanism of sorption: direct sorption, which corresponds to the water molecules that form hydrogen bonds directly with toCNF, and indirect sorption, which corresponds to water molecules that bind with already bound water molecules [10]. Interfibril interactions, on the other hand, may inhibit swelling [60], because the increase in volume of hydrated nanofibrils can be slowed down by other nanofibrils and water binding to the nanofibrils reduces the interfibril binding. While adsorption is a fast process, absorption occurs more slowly, with less water molecules penetrating the inner surface and amorphous regions [61]. SEM images of the cross-section of films (Figure 3) show the same laminar structure with a similar density, regardless the carboxyl content and casting conditions.

In addition, the sorption equilibrium was achieved within a relatively short period of time, with about 65% of the sorption equilibrium reached after 30 min for films cast in acidic conditions, up to around 80% for films containing cyclodextrins. The difference in sorption equilibrium observed between L-toCNF and H-toCNF can be linked to the number of carboxylic functions prone to form H-bonds with water molecules, which is higher for H-toCNF than for L-toCNF. The increase in sorption for films prepared under acidic conditions is explained by the acid form of the carboxylic groups, which is more prone to form H-bonds with water molecules than the carboxylate form at neutral pH. Finally, the decrease in sorption with the addition of cyclodextrin is thought to be due to the adsorption of β-CD on the surface of the fibres, which could decrease the amount of water bounded to the fibres. Water sorption is mainly driven by adsorption and chemical makeup-dependent. For a given drying temperature, the water sorption can be slightly tuned by varying the process parameters, which is an interesting property for drug delivery applications, where swelling and sorption properties are important for the delivery kinetics.

#### 2.1.2. Cryogels

**Figure 4:**
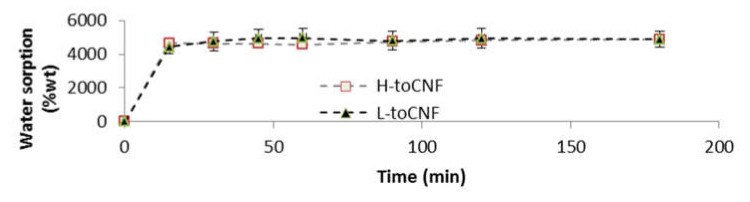
Water sorption for toCNF cryogels in immersion.

Water sorption tests for cryogels were conducted gravimetrically. The cryogels were weighted and immersed in distilled water and then removed at different time intervals. Tissue paper was used to remove excess water prior to weighing. For both charge contents, the swelling equilibrium was reached after the first measurement at 15 min, as shown in Figure 4. The swelling equilibrium after 3 h was 4871 ± 471% and 4890 ± 141% for L-toCNF and H-toCNF samples, respectively. According to these results, cryogels were immersed 1 h before compression to study the mechanical properties of swollen cryogels so that they would be at swelling equilibrium. It also appeared that the charge content does not have a significant impact on the sorption equilibrium, suggesting that the absorption mechanism in immersion is mainly driven by the porosity and the pore morphology, which are not strongly affected by the charge content.

### 2.2. Mechanical Characterisation

Compression tests were carried out on cryogels of cylindrical shape. The density of each sample was determined by dividing the mass of each cryogel by its volume. The volume of the cryogels was measured from height and diameter measurements using a calliper. In each case, the compression curve can be divided into three different regions: For low-strain values, the compression stress increases linearly with the strain in the elastic domain up to the yield point. The compression modulus was calculated at a strain corresponding to half the yield stress in order to be reproducible between all samples. For strains higher than the yield point, the plastic region was reached. In this region the stress increases with the strain with significant residual deformation after unloading. For high compressive strains, the curves exhibited a sharp increase in the compressive stress, typical of a densification regime.

**Figure 5:**
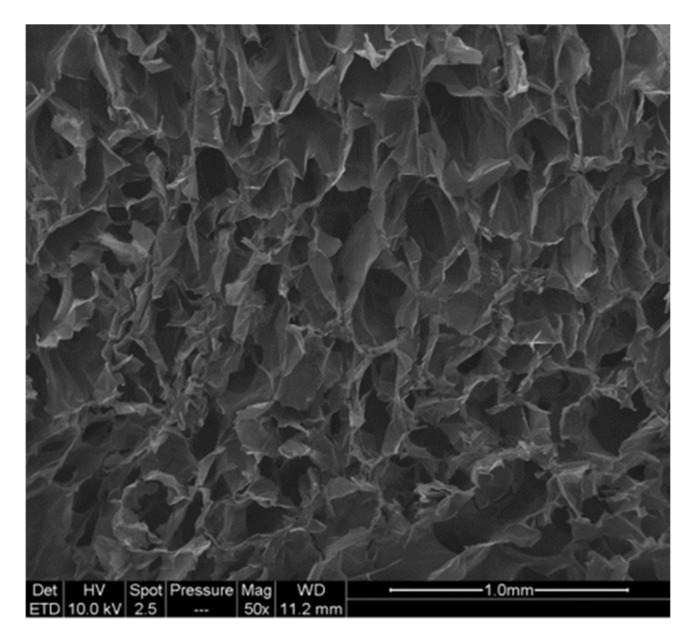
SEM images of the cross-section of toCNF cryogel.

This behaviour has previously been reported for cellulose-based foam materials [15,16,18,19]. The cryogels obtained are closed-cell wall foams, as observed in Figure 5. In such materials, elasticity is caused by the stretching of the cell walls, plastic deformation occurs when the cells are starting to lose their integrity, and densification occurs when cells collapse on themselves, reducing porosity and causing the cryogels to behave like the solid itself [62,63].

#### 2.2.1. Impact of Density

**Figure 6:**
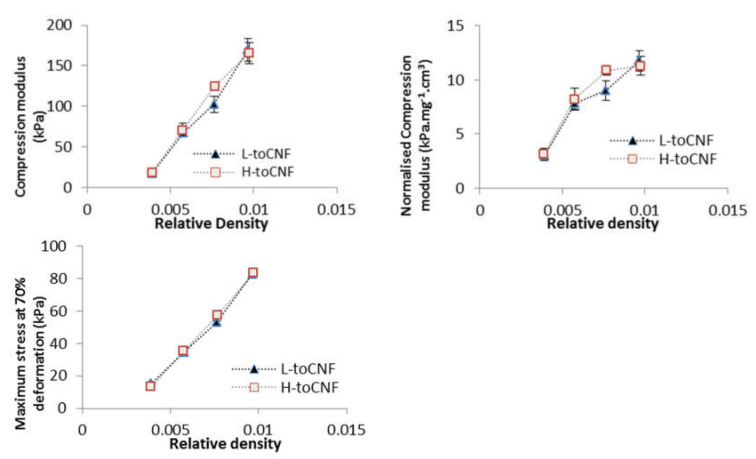
Relative density dependence of the compression modulus, normalised compression modulus, and maximum stress at 70% deformation for L-toCNF and H-toCNF cryogels.

Compression tests were carried out on cryogels with four different densities prepared from toCNF with both charge densities. Variations of the different properties (compression modulus, normalised compression modulus, and maximum stress at 70% deformation) with the relative density are presented in Figure 6, and numerical values are summarised in Table 4. The results for L-toCNF and H-toCNF are similar for all densities, indicating that the charge content has no major impact on the mechanical properties under the process conditions tested. All the properties increased with the density, quite linearly for the compression modulus, and the maximum stress at 70% deformation, while the normalised compression modulus seems to stabilise for densities higher than 15 mg/cm^3^ (which corresponds to a relative density of 0.01). It is worth noting that the normalised compression modulus withstands a huge decrease for relative densities lower than 0.008. Density is of major importance for the mechanical behaviour of cryogels, and by controlling the density, it is possible to tailor the mechanical properties of the cryogel.

#### 2.2.2. Impact of pH and Cyclodextrins on Dry Cryogels

**Figure 7:**
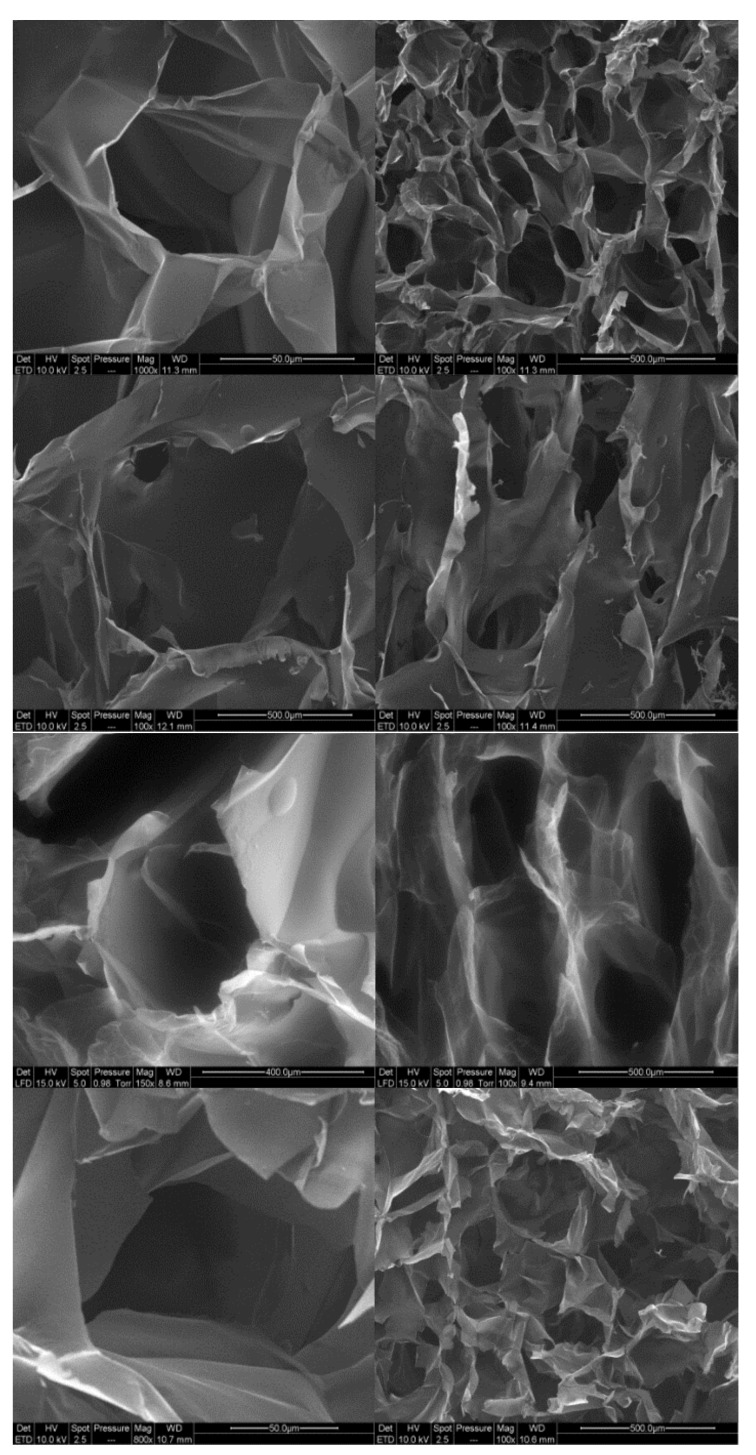
SEM images for toCNF cryogels. From Top to bottom: cryogels from suspensions 1/2/3/4.

Cryogels were prepared from the four compositions by freeze-drying the nanofibril suspensions. SEM images of the cross-section of the cryogels are presented in Figure 7. For all cryogels, closed-cell wall structures are observed, organised as an alveolar structure.

This specific orientation of porosity is due to the freeze-drying process. Indeed, freezing occurred from the bottom part of the freeze-dryer, resulting in the growth of ice in a specific direction, leading to anisotropy in the pore orientation.

**Figure 8:**
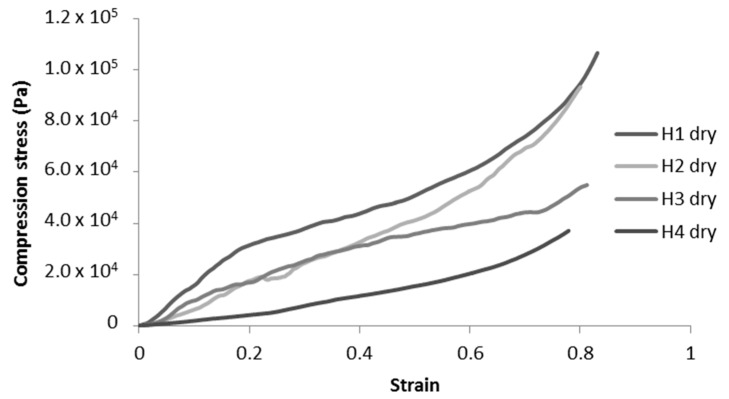
Representative compression curves for H-toCNF cryogels.

In addition, we can observe that the structure of the cell walls for modified toCNF cryogels presents more structural deflects (holes and folds) than the unmodified toCNF one. Compression tests were carried out on cryogels prepared from the four compositions for both charge contents. Typical stress-strain curves for each composition are presented in Figure 8.

Unmodified toCNF cryogels (H1 and H2) exhibit clear elastomeric behaviours, with well-defined linear elastic zones up to a strain of about 0.2, followed by a compression plateau and a densification for higher strains. For modified toCNF cryogels (H3 and H4), the linear elastic zone is restrained to lower strain values, and the yield point is less marked. The deflects observed in Figure 7 in the cell walls for modified toCNF cryogels oppose the elastic buckling of the cells, explaining the small elastic region for modified cryogels. The density, compression modulus, normalised compression modulus, and maximum stress at 70% deformation for each type of cryogel and for both charge contents are summarised in Table 5.

**Figure 9:**
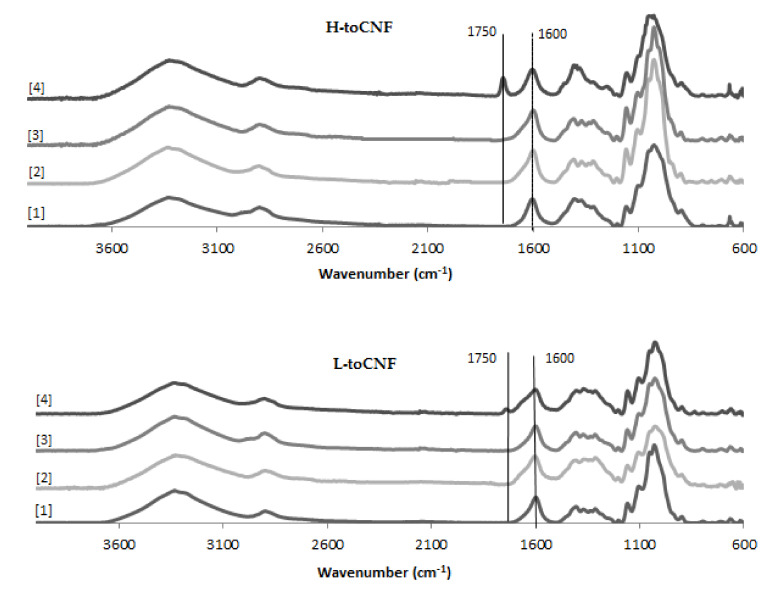
Attenuated total reflectance-Fourier transform infrared (ATR-FTIR) spectra for toCNF cryogels.

An increase in density can be observed for cryogels cast under acidic pH and with cyclodextrins. The carboxyl content increases the interactions between the fibres in its carboxylic form, as it forms a more densely packed structure. The adsorption of cyclodextrins on the surface of the fibres also increases the interaction between fibres or creates local deflects in the fibres’ arrangement. Both mechanisms impact the mechanical properties. The mechanical properties decrease when the density increases, and the elastic region in the stress-strain curve also decreases for cryogels cast under acidic pH or when containing cyclodextrins. It is also worth noting that the yield point is lower for modified cryogels (pH and cyclodextrins) than for the unmodified one and that the plateau is less pronounced. Since elasticity of foams is linked to the stretching of the cell walls and the plastic behaviour prior to densification is linked to the compression of cells, the modification of fibre-fibre interactions under acidic pH and/or with adsorption of cyclodextrin on the fibre surface is responsible for the modification of the cell wall, thus the mechanical properties. Both charge contents exhibit a similar behaviour for toCNF, toCNF pH 2.5, and toCNF 10 wt% CD, but a noticeable difference is observed for toCNF 10 wt% CD pH 2.5. For L-toCNF, the density decreases between pH 2.5 and 10 wt% CD pH 2.5, while the normalised compression modulus and the maximum stress at deformation are quite similar. For H-toCNF, the density increases between pH 2.5 and 10 wt% CD pH 2.5, while the normalised compression modulus and the maximum stress at 70% deformation decrease. Under these conditions (10 wt% CD and pH 2.5), esterification occurred between the carboxylic acid of the toCNF and hydroxyl groups of cyclodextrins, as evidenced by the attenuated total reflectance-Fourier transform infrared (ATR-FTIR) spectra shown in Figure 9.

The peak observed at 1600 cm^−1^ corresponds to the carboxylate ions present on the surface of the fibres introduced during TEMPO-mediated oxidation. For toCNF 10wt% CD pH 2.5, a peak at 1750 cm^−1^ can be observed which corresponds to the ester groups. The presence of this esterification peak only for toCNF 10wt% CD pH 2.5 indicates that the esterification reaction occurred between the hydroxyl groups of the cyclodextrins and the carboxylic acid groups of toCNF under acidic pH, suggesting that the lyophilisation process allows the reaction by the removal of water. Considering the respective charge contents for L-toCNF and H-toCNF, the efficiency of esterification might be higher for H-toCNF, which can explain the decrease in mechanical properties observed for H4.

#### 2.2.3. Difference Between Dry and Swollen Cryogels

**Figure 10:**
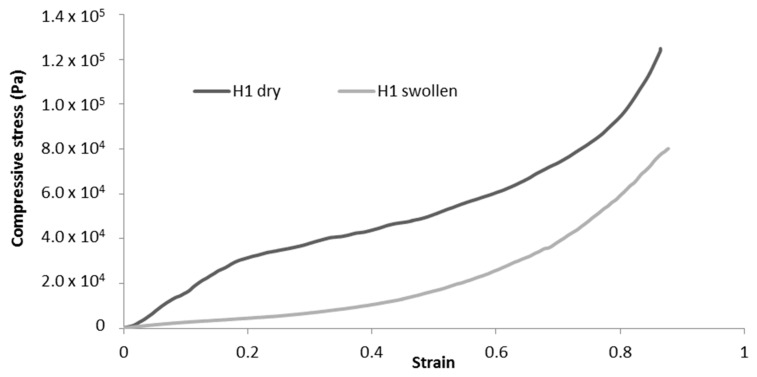
Representative compression curves for dry and swollen H-toCNF cryogels.

Cryogels were immersed in water for 1 h prior to the experiment, and tissue paper was used to remove excess water before compression. The changes in mechanical properties between the dry and swollen states are summarised in Table 6. Comparison of the typical stress-strain curves for dry and swollen cryogels are presented in Figure 10. For swollen cryogels, no clear elastic zone is observed. The water molecules, by binding to the fibrils, inhibit interfibril interactions and, thus, largely reduce the elastic behaviour. As a result, a significant decrease in mechanical properties is observed for each sample tested, as illustrated in Table 6.

Since no clear elastic deformation zone can be observed for swollen cryogels, the compression modulus was calculated at a low strain (< 0.1) for comparison with dry cryogels. The decrease in mechanical properties is more significant for H-toCNF (H1, H2, and H3 cryogels), which can be explained by the higher charge content, making the fibres more sensitive to the adsorption of water molecules. It is also worth noting that the decrease in mechanical properties is reduced for H4 in comparison with L4, with respectively −49% and −77% decreases in the compression modulus compared to their dry state. This behaviour could be explained by the esterification between cyclodextrin and toCNF, cross-linking fibres, and making the structure less sensitive to water ad/absorption. 

Esterification of βCD with toCNF under acidic conditions by freeze-drying was proven. Nevertheless, some questions remain about the yield of grafting and the adsorption mechanism between unbound cyclodextrins and toCNF. Given the chemical similarity between toCNF and βCD, direct characterisation and quantification of grafting seems impossible, as a cyclodextrin with several hydroxyl functions is likely to bind with the carboxylic acid of toCNF. Nevertheless, the presence of multiple hydroxyl functions on both toCNF and βCD indicates a strong adsorption between these two components. However, some cyclodextrins might be trapped in the ice phase during the freezing process and, therefore, are not available for an esterification reaction during the freeze-drying phase. In order to better quantify this adsorption and to be able to confirm that no material will be released from the materials produced, further studies, in particular with the means of Quartz Crystal Microbalance with Dissipation monitoring (QCM-D) and Isothermal Titration Calorimetry (ITC), will be conducted. Additionally, a method adapted from [56] will be implemented, with the use of phenolphthalein (PhP). The interactions between PhP and βCD, described by the authors of [64], could lead to an indirect estimation of βCD available in the materials, and released measurements of βCD could lead to an estimation of the portion of cyclodextrins linked (adsorbed/grafted) to toCNF.

The compression modulus for swollen modified toCNF cryogels ranges between 6 and 9 kPa, i.e., in the range of mechanical stiffness of muscle tissue, and the high porosities obtained (> 99% for all conditions), which are mandatory to promote a good vascularisation, the diffusion of nutrients to the cells, and tissue growth, made this structurally suited for tissue engineering applications. Further studies will be focused on the mechanical properties of such materials under successive stress. Nevertheless, to confirm the potential for tissue-engineering applications, cytotoxicity and degradation studies need to be done in further work. However, βCD toCNF materials could be of a great interest for other applications, such as filtration or depollution, using cyclodextrins to capture molecules of interest rather than release them.

## 3. Materials and Methods 

### 3.1. Materials

A mixture of bleached and never-dried spruce (picea abies ca. 75%) and pine (pinus sylvestries, ca. 25%) cellulose pulp from Södra (Växjö, Sweden) was used as raw material. All chemicals used in this study were of laboratory-grade quality purchased from Sigma-Aldrich, St. Louis, MO, USA.

### 3.2. Preparation of toCNF With Two Different Charge Contents

TEMPO-oxidised cellulose nanofibrils were produced according to a protocol adapted from [6]. Never-dried cellulose (110 g of cellulose content) was suspended in water (3 L) and stored overnight at 4 °C. The suspension was dispersed with a blender and mixed with a solution (400 mL) containing TEMPO (1.375 g) and sodium bromide (13.75 g). Water was added to obtain a total volume of 8250 mL (75-mL/g cellulose). TEMPO-mediated oxidation of cellulose was started by adding different amounts of 13% NaClO: 2.5 mmol/g cellulose for a charge content of 750 µmol/g and 3.3 mmol/g cellulose for a charge content of 1100 µmol/g. NaClO were added gradually, and the pH was maintained at 10.5 by adding 0.5M NaOH. The slurry was stirred for 15 min after the complete addition of NaClO, and the pH was then dropped to 7 with 0.1-M HCl. Methanol (100 mL) was then added to the slurry. The product was thoroughly washed with water by filtration until the conductivity of the filtrate was below 5 µS/cm. Homogenisation was conducted using a Rannie 15 type 12.56 × homogeniser (APV, SPX Flow Technology, Silkeborg, Denmark). The suspension was diluted to 1.2 wt% and dispersed with an electric mixer. The fibres underwent two passes in the homogeniser at 600 bar and 1000 bar, respectively. The final suspensions were stored at 4 °C.

### 3.3. Determination of the Charge Content

The carboxyl group content was determined by conductometric titration as described in previous studies (e.g., [7,32,65]). NaCl (5 mL 0.1M) was added to a toCNF dispersion with 0.2-g solid content in 450 mL. The pH was adjusted to approximately 2.5 by addition of 0.1-M HCl and further diluted with water to a total volume of 500 mL. The dilution was titrated with 0.05-M NaOH solution added at a rate of 0.15 mL/min under stirring up to a pH of 11. An automatic titrator (902 Titrando, Methrom AG, Herisau, Switzerland) was used, and the conductivity of the sample was automatically measured (856 Conductivity Module, Methrom AG, Herisau, Switzerland) for increments of 0.02 mL. Data were recorded by Tiamo Titration software. The carboxyl content was calculated from the titration curve using the Gran plot. Duplicates were made for both suspensions and NaOH titration (control).

### 3.4. Material Processing 

#### 3.4.1. Film Processing

Dry toCNF (0.25 g) was weighed and diluted with water to a total volume of 50 mL. The suspension was dispersed for 2 min at 7000 rpm with an UltraTurrax (IKA-Werke, Staufen, Germany), at room temperature. βCD (0.025 g) and 0.1-M HCl (2 mL) were added to the relevant samples. The suspensions were magnetically stirred for 1 h and placed in an ultra-sonic bath for 3 min. The suspension was then cast in petri dishes (9-cm diameter) and stored in an oven at 40 °C for 18 h. The resulting films were stored in closed petri dishes at room temperature.

#### 3.4.2. Cryogel Processing 

Impact of pH, cyclodextrin, and comparison dry/swollen: Fifty millilitres of 0.8 wt% toCNF suspensions were prepared and dispersed 2 min at 7000 rpm with an UltraTurrax. βCD (0.04 g) and 0.1-M HCl (3 mL) were added if required. The suspensions were magnetically stirred for 1 h and placed in an ultra-sonic bath for 3 min. The suspensions were poured into a 24-well plate (3 mL per well) and freeze-dried for 24 h at −20 °C and 0.3 mbar (BK FD12S, Biobase Biodustry, Jinan, China). The resulting cryogels were stored in closed well plates.

Impact of density: Fifty millilitres of L-toCNF and H-toCNF suspensions at 1 wt%, 0.8 wt%, 0.6 w%, and 0.4 wt% were prepared and dispersed for 2 min at 7000 rpm with an UltraTurrax. The suspensions were magnetically stirred for 1 h and placed in an ultra-sonic bath for 3 min. The suspensions were then poured into a 24-well plate (3mL per well) and put in a freezer at −20 °C for 24 h before freeze-drying (ALPHA 2-4 LDplus, Christ ^®^, Osterode am Harz, Germany).

### 3.5. Water Sorption Analysis

Water sorption tests on films were carried out gravimetrically in a Percival climatic chamber at 25 °C and 90% RH (relative humidity). The samples were weighted every hour at the beginning of the experiment and at selected times thereafter. Water sorption experiments were conducted after 48 h, with at least 3 replicates for each sample. The samples were put in a desiccator for 16 h prior to the experiment. The water sorption was characterised by the weight change between the initial sample weight (m_0_) and the weight after a certain time t (m_t_), according to Equation (1):

(1) $$Water\;sorption\;\left[\%\right]=\frac{m_{t}-m_{0}}{m_{0}}\times 100$$

Water sorption test on cryogels were conducted gravimetrically. The cryogels were weighted and immersed in distilled water and then removed at different times. Excess water was removed before weighting. Water sorption was calculated using Equation (1).

### 3.6. Microscopy

Atomic force microscopy images were recorded on a Dimension icon^®^ (Bruker, Billerica, MA, USA). The concentration of the suspension was adjusted to 10^−3^ wt% by diluting the CNF dispersion using the high shear mixer Ultra-Turrax. A drop of this suspension was deposited on a freshly cleaved mica plate before drying overnight under a fume hood at room temperature. The acquisition was performed in tapping mode using a silica-coated cantilever (OTESPA^®^ 300 kHz-42 N/m, Bruker, Billerica, MA, USA). Zones of 1.1*1.1 μm^2^ were analysed.

Scanning electron microscopy images were performed with ESEM (Quanta 200, FEI, Japan). Film and cryogel cross-sections were cut with a razor blade. SEM observation was carried out on cross-sections after carbon sputter coating of 5 nm, with a tension of 10 kV and a spot size of 3.5. The working distance was set between 9.5 mm and 11.5 mm depending on the sample.

For both microscopy techniques, at least 5 different images were performed to check the consistency in various zones of the sample, and the most representatives were selected for the discussion.

### 3.7. Mechanical Characterisation 

Impact of density: Compression tests were performed using a TA Instruments RSA 3 (New Castle, DE, USA) dynamic mechanical analyser fitted with a 100-N load cell. Samples prepared as cylinders were individually measured and compressed with a crosshead speed of 0.1 mm/s at room temperature. At the least, triplicates were performed, and the average is presented.

Impact of pH, cyclodextrin, and comparison dry/swollen: Compression tests were performed with a Stable Micro Systems TA-XT2 texture-analyser (Stable Micro Systems, Godalming, UK), equipped with a P/35 probe and with a crosshead speed of 0.1 mm/s, as previously described by Heggset et al., 2018 [66].

A minimum of 6 cryogels were tested for each sample. The compression modulus was calculated in the elastic region at half the strain of the beginning of the plateau region, and the stress at 70% strain was directly read from the data. The normalised compression modulus was calculated by dividing the compression modulus by the cryogel density. The cryogel density ρ was determined by dividing the mass of each cryogel by its volume. The volume of produced cryogels was measured from height and diameter measurements using a calliper. For each sample, the two extreme values were removed. The relative density of the cryogels was calculated from the ratio ρ/ρ_c_, where ρ_c_ is the density of cellulose, 1.5 g/cm^3^ [67]. The porosity was calculated from Equation (2):

(2) $$Porosity\;\left[\%\right]=\left(1-\frac{\rho }{\rho_{C}}\right)*100$$

### 3.8. Fourier Transform Infrared Spectroscopy

Infrared spectra were recorded in attenuated total reflectance (ATR) mode, using a Perkin Elmer Spectrum 65 (Perkin Elmer, Wellesley, MS, USA). Spectra were recorded between 4000 and 600 cm^−1^, with 16 scans and a resolution of 4 cm^−1^. Since this technique is used to determine the possible esterification between the cyclodextrins and the toCNF, and given the proximity between the carboxylic peak and the ester peak (respectively, ≈1720 cm^−1^ and ≈1750 cm^−1^), each cryogel was dipped in 0.05 M NaOH for 10 s to convert carboxylic acid groups to carboxylate groups (1600 cm^−1^) and dried in the oven for 30 min prior to analysis. As the control sample, the neat samples and neat samples after 30 min drying in the oven were also analysed to ensure that esterification was only due to the freeze-drying process. At least 5 different zones of the sample were analysed, and the most representative spectra were used for discussion.

## 4. Conclusions

In this work, films and cryogels of β-cyclodextrin-modified TEMPO-oxidised cellulose nanofibrils were produced. Water sorption analysis and mechanical characterisation were conducted on both modified and unmodified materials under dry and wet conditions. Two unmodified nanofibrils suspensions were prepared with different charge contents (750 µmol/g and 1050 µmol/g), and modification was carried out under neutral and acidic conditions. The sorption equilibrium was reached after 4 h for all films tested, but the charge content and acidic casting pH were shown to increase the water sorption, while cyclodextrins decreased it. Density, process pH, and the addition of cyclodextrins had major impacts on the mechanical properties, related to the modification of the cell wall structure. Finally, covalent esterification binding between β-cyclodextrin and toCNF under acidic pH by freeze-drying was achieved and had an interesting impact on the mechanical properties in the swollen state. This study is a step towards the production of mechanically tailored cryogels containing cyclodextrin, making them promising materials for the sustained delivery of active principle ingredients.

## Figures and Tables

**Table 1:** Previous works on the association nanocellulose-cyclodextrin. CNFs: cellulose nanofibrils, toCNFs: TEMPO-oxidised cellulose nanofibrils, HP-CNFs: hydroxypropyl cellulose nanofibrils, CNCs: cellulose nanocrystals, βCD: β-cyclodextrin, CMβCD: carboxymethyl- β-cyclodextrin, HPβCD: hydroxypropyl-β-cyclodextrin.

Nanocellulose	CD	Functionalisation Strategy	Application	Source
toCNFs	βCD	Direct grafting	Release of essential oil	[48]
CNFs	βCD	Cross-linking with citric acid	Depollution	[49]
toCNFs	CMβCD	Amidation via EDC/NHS	Depollution	[50]
HP-CNFs	HPβCD	Electrospinning	Drug release	[51]
CNFs	βCD	Coating/Adsorption	Drug release	[52]
CNFs	βCD	Cross-linking with citric acid	Antibacterial packaging	[53]
toCNF	βCD	Noncovalent interaction	Drug Delivery/ Tissue Engineering	[29]
toCNCs	βCD/HPβCD	Direct grafting	Release of essential oil	[54]
CNCs	βCD	Grafting with epichlorohydrin	Tissue engineering	[55]
CNCs	βCD	Crosslinking with fumaric and succinic acid	Release of essential oil	[56]
CNCs	βCD	Ionic interaction	Drug delivery	[57]
CNCs	βCD	Grafting with epichlorohydrin	Supramolecular hydrogels	[58]

**Table 2:** Samples codes.

Sample Code	βCD	pH
1	0	Neutral
2		Acidic
3	10 wt%	Neutral
4		Acidic

**Table 3:** Sorption equilibrium after 48 h and % of sorption after 30 min for low-charge content cellulose nanofibrils (L-toCNF) films and high-charge content cellulose nanofibrils (H-toCNF) films.

Sample	Water Sorption Equilibrium after 48 h (wt%)		% of Sorption after 30 min	
	L-toCNF	H-toCNF	L-toCNF	H-toCNF
1	21.5 ± 0.2	23.2 ± 0.2	77.6 ± 0.2	76.7 ± 1.2
2	28.3 ± 0.3	32.3 ± 0.6	69.3 ± 2.0	62.9 ± 2.1
3	18.6 ± 0.1	19.0 ± 0.4	82.0 ± 1.5	83.9 ± 0.9
4	25.2 ± 0.5	27.1 ± 0.5	67.9 ± 2.0	74.1 ± 0.5

**Table 4:** Mechanical properties for toCNF at various densities.

Initial Dry Content	Porosity (%)		Compression Modulus (kPa)		Normalised Compression Modulus (kPa·mg^−1^·cm^3^)		Maximum Stress at 70% Deformation (kPa)	
	L-toCNF	H-toCNF	L-toCNF	H-toCNF	L-toCNF	H-toCNF	L-toCNF	H-toCNF
0.4 wt%	99.6	99.6	17 ± 2	18 ± 3	3.0 ± 0.3	3.1 ± 0.6	15 ± 1	13 ± 1
0.6 wt%	99.4	99.4	68 ± 5	71 ± 9	7.8 ± 0.6	8.2 ± 1.0	35 ± 1	35 ± 1
0.8 wt%	99.2	99.2	102 ± 10	125 ± 4	9.0 ± 0.9	10.9 ± 0.4	53 ± 1	57 ± 2
1 wt%	99.0	99.0	170 ± 14	165 ± 13	11.8 ± 0.9	11.3 ± 0.9	83 ± 1	84 ± 1

**Table 5:** Mechanical properties of to-CNF cryogels of different compositions.

	Cryogel Density (mg/cm^3^)		Compression Modulus (kPa)		Normalised Compression Modulus (kPa·mg^−1^·cm^3^)		Maximum Stress at 70% Deformation (kPa)	
	L-toCNF	H-toCNF	L-toCNF	H-toCNF	L-toCNF	H-toCNF	L-toCNF	H-toCNF
1	14.89 ± 0.47	16.07 ± 0.57	104 ± 13	150 ± 10	7.0 ± 0.8	9.4 ± 0.8	64 ± 3	65 ± 3
2	25.32 ± 0.84	20.82 ± 1.08	34 ± 6	76 ± 6	1.4 ± 0.2	3.7 ± 0.3	58 ± 7	63 ± 3
3	17.91 ± 0.60	18.45 ± 1.66	59 ± 10	84 ± 13	3.3 ± 0.5	4.7 ± 0.3	42 ± 2	43 ± 2
4	20.85 ± 0.96	26.41 ± 2.19	37 ± 3	17 ± 3	1.8 ± 0.2	0.6 ± 0.1	59 ± 5	24 ± 4

**Table 6:** Diminution of mechanical properties between the dry and swollen states.

	Compression Modulus (kPa)		Diminution of Compression Modulus (%)		Diminution of Maximum Stress at 70% Deformation (%)	
**Casting Conditions**	L-toCNF	H-toCNF	L-toCNF	H-toCNF	L-toCNF	H-toCNF
1	45 ± 4	33 ± 4	−57 %	−78%	−4%	−54%
2	9 ± 1	8 ± 1	−75 %	−90%	−82%	−90%
3	6 ± 1	7 ± 1	−89%	−92%	−90%	−89%
4	9 ± 1	9 ± 4	−77%	−49%	−82%	−73%
